# Unveiling readability challenges: An extensive analysis of consent document accessibility in clinical trials

**DOI:** 10.1017/cts.2024.595

**Published:** 2024-09-16

**Authors:** Adrian H. Zai, Jamie M. Faro, Jeroan Allison

**Affiliations:** Department of Population and Quantitative Health Science, University of Massachusetts Chan Medical School, Worcester, MA, USA

**Keywords:** Consent forms, comprehension, informed consent, health literacy, recruitment

## Abstract

**Background:**

Clinical research trials rely on informed consent forms (ICFs) to explain all aspects of the study to potential participants. Despite efforts to ensure the readability of ICFs, concerns about their complexity and participant understanding persist. There is a noted gap between Institutional Review Board (IRB) standards and the actual readability levels of ICFs, which often exceed the recommended 8th-grade reading level. This study evaluates the readability of over five thousand ICFs from ClinicalTrials.gov in the USA to assess their literacy levels.

**Methods:**

We analyzed 5,239 US-based ICFs from ClinicalTrials.gov using readability metrics such as the Flesch Reading Ease, Flesch-Kincaid Grade Level, Gunning Fog Index, and the percentage of difficult words. We examined trends in readability levels across studies initiated from 2005 to 2024.

**Results:**

Most ICFs exceeded the recommended 8th-grade reading level, with an average Flesch-Kincaid Grade Level of 10.99. While 91% of the ICFs were written above the 8th-grade level, there was an observable improvement in readability, with fewer studies exceeding a 10th-grade reading level in recent years.

**Conclusions:**

The study reveals a discrepancy between the recommended readability levels and actual ICFs, highlighting a need for simplification. Despite a trend toward improvement in more recent years, ongoing efforts are necessary to ensure ICFs are comprehensible to participants of varied educational backgrounds, reinforcing the ethical integrity of the consent process.

## Background

In clinical research, informed consent forms (ICFs) embody a critical juncture in the translational science continuum, facilitating the conveyance of complex information from researchers to participants. These documents are pivotal in regulatory compliance and essential in bridging the gap between scientific discovery and patient care. Achieving a balance in ICFs – where they are comprehensive enough to include detailed medical and research-related information yet sufficiently accessible for nonspecialist audiences – is a challenge that lies at the heart of ethical research practices and translational science. This duality recognizes the imperative of simplifying intricate concepts without diluting the depth and accuracy necessary for informed consent, a cornerstone in enhancing patient understanding and potential ability to increase adherence to study requirements.

The readability of ICFs is a crucial aspect of this process, significantly influencing a participant’s ability to make truly informed decisions. This challenge reflects a broader issue in translational science: the need to communicate complex scientific concepts in an accurate and understandable manner to the general public. While readability indices offer a measure of text accessibility, comprehension remains the ultimate goal to ensure informed consent, as it is a step toward building trust in research and between participants and the research team.

Historical evaluations, such as those by Paasche-Orlow *et al*. (2003), have highlighted a significant discrepancy between the readability standards recommended by Institutional Review Boards (IRBs) – typically an 8th-grade reading level and shorter length ICFs [1,2], which are recommendations rather than strict regulations – and the actual complexity of consent forms. This gap poses a potential barrier to participant understanding and informed consent, underscoring the importance of addressing this challenge within the context of translational science [3]. Further studies, including those conducted in South Africa, found that the average reading level of ICFs corresponded to the 12th grade, with a minuscule percentage written at the recommended 8th-grade level, emphasizing the global relevance of this issue [4]. These and similar findings from various studies [5–12] underscore the challenge of achieving readability and comprehension in ICFs.

This paper aims to deepen our understanding of the literacy demands of ICFs by conducting an exhaustive review of over five thousand ICFs for clinical trials conducted in the USA and listed in ClinicalTrials.gov [13]. This comprehensive analysis markedly diverges from the prevailing literature, which often derives conclusions from considerably smaller datasets, typically examining fewer than 200 ICFs. Such restricted scopes frequently limit their focus to specific clinical phases [5], diseases [14], procedures [15], or geographic locations [16], thereby providing insights that, while valuable, do not capture the full spectrum of readability challenges across the broader landscape of clinical research.

By adopting a broad lens, this study seeks to uncover the extensive readability challenges that pervade ICFs across various clinical trials. This approach allows us to extend beyond the limitations of studies with smaller sample sizes and also report on trends over time. This is particularly meaningful as the research community has long recognized the need to improve readability, and our results show we are not there yet. In doing so, it aspires to offer insights crucial for advancing informed consent processes, thereby reinforcing translational science principles.

## Methods

### Data collection

**Figure 1.**
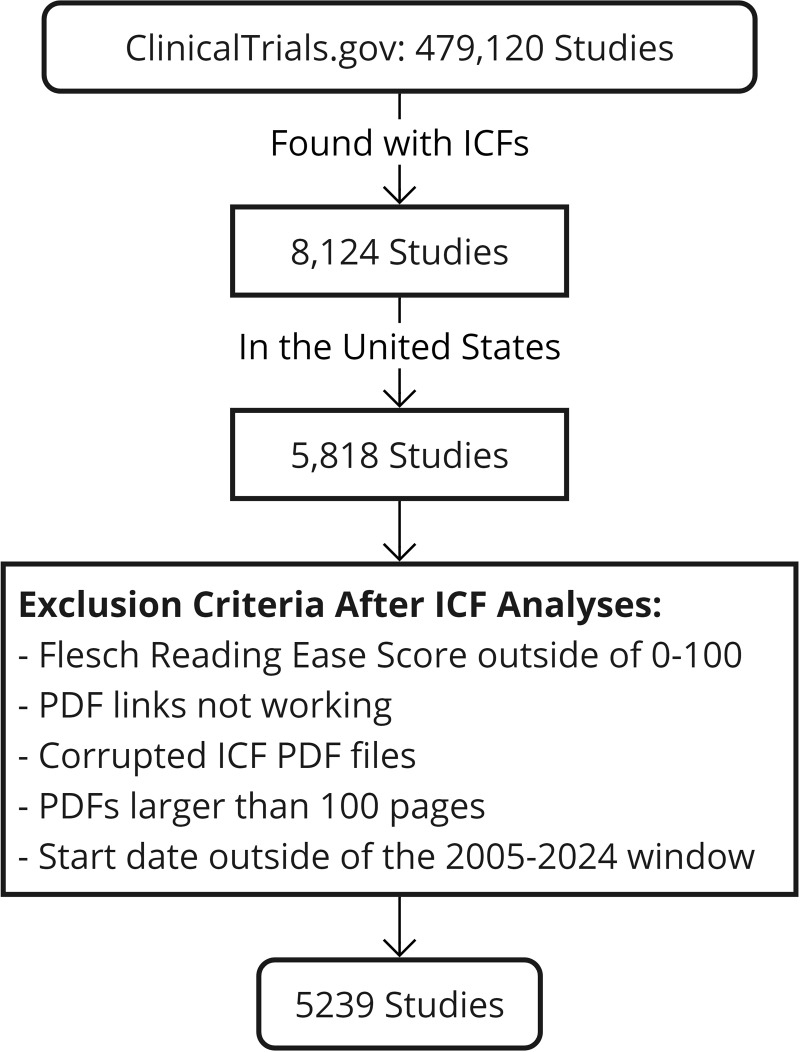
Flow diagram of study selection process. ICF = informed consent form.

Our research started with a targeted search on ClinicalTrials.gov, conducted on January 20, 2024, with the specific objective of identifying clinical studies within the USA that included ICFs for studies conducted between 2005 and 2024 (Figure 1). This geographical focus was necessary to ensure the analysis was confined to ICFs in English, thereby avoiding the complexities associated with ICFs in foreign languages. From the 479,120 studies indexed on the platform, we applied a filter for US-based studies with ICFs, which resulted in identifying 5,818 studies that met our criteria. This subset was selected to represent a comprehensive cross section of clinical trials across various medical fields, providing a focused and relevant sample for our analysis of ICF readability within the USA. Additionally, the studies included all age groups. For pediatric studies, we analyzed the consent form given to parents only.

### Data cleaning

A data cleaning step was implemented to ensure our dataset’s integrity and quality. Our initial step involved the removal of clinical trials that lacked start dates or had associated ICF PDF files that were missing or corrupted. This measure was essential to preserve the accuracy and relevance of our study. When specific dates within the dataset only included month and year, we standardized these by setting the missing days to the first of the respective month.

The dataset underwent further refinement to enhance its quality. Key among these was the exclusion of ICFs with zero-word counts, which typically resulted from unsuccessful conversions of PDF documents into electronic readable text. We also removed ICFs with Flesch Reading Ease scores outside the normal range of 0–100, as such anomalies in scores often reflect issues in the text’s structure or content. For example, ICFs containing an excessive number of tables often scored over a hundred because short sentences in tables would skew the readability assessments. Another criterion for exclusion was the length of the PDFs; those exceeding 100 pages were often part of extensive protocols or combined with academic papers, necessitating their removal to maintain our focus on the consent forms’ content only. We excluded studies with start dates outside the 2005 to 2024 window, as their limited numbers could skew our trend analysis. These data cleaning steps led to a refined dataset of 5,239 ICFs, as detailed in Table 1.

**Table 1.** Characteristics clinical trials informed consent forms (ClinicalTrials.gov, 2005–2024, *n* = 5239)

	ICF count
Total	5239
Funder type	
Industry	342
Network	42
National Institutes of Health	323
Federal	245
Other	4287
Enrollment	
Only male	205
Only female	491
Both	4541
Unknown	2
Age	
Only children	321
Only older adults	85
Adult+older adults	3913
All	287
Study type	
Observational	418
Interventional	4821

### Data extraction and transformation

In the data extraction and transformation phase, we employed a Python script with the PyPDF2 module v.3.0.1 [17] for automated text extraction from PDF files of ICFs. PyPDF2, chosen for its adeptness in handling PDFs in Python, was instrumental in this process [18–20].

The script initiates by opening the ICF PDFs with PyPDF2’s PdfFileReader function, effectively managing text layers and embedded fonts. It then systematically extracts text from each page using the getPage and extractText methods. This approach efficiently parses and converts PDF text layers into string format.

An essential transformation step consolidates the extracted text into a continuous format, enhancing its suitability for analysis. PyPDF2’s capability to manage complex PDF elements like varied text alignments and embedded tables ensures accurate text extraction.

Integrating PyPDF2 significantly streamlines the extraction process, saving time and minimizing manual errors, thus enhancing the reliability and accuracy of the data extraction. This method effectively transforms ICF PDF documents into an analyzable format, demonstrating the robustness of our Python-based approach.

### Page count and text analysis

The initial step in our analysis involved counting the total number of pages for each ICF. This provided an overview of the IPF documents’ length and potential complexity, an essential factor in assessing the readability of the ICFs.

### Readability metrics

We employed three established readability algorithms to thoroughly assess the readability of ICFs, each offering unique insights [21,22]. These three indices together help gain a comprehensive view of the readability of ICFs, allowing for a multifaceted approach to enhancing their clarity and accessibility. These algorithms are available in the Python textstat library version 0.7.3 [23].

The Flesch Reading Ease [24] metric is designed to assess the readability of texts by considering two key factors: the average length of sentences and the average number of syllables per word. It calculates scores ranging from 0 to 100, where higher scores indicate texts that are easier to read. The formula for this metric involves a specific calculation: 206.835 − (1.015 × average sentence length) − (84.6 × average syllables per word). The Flesch Reading Ease metric, focusing on sentence length and syllable count per word, quantitatively evaluates how easily a text can be read. This metric is beneficial in identifying overly complex word usage and long sentences that could hinder comprehension.

The Flesch-Kincaid Grade Level [24], closely related to the Flesch Reading Ease, provides an estimated US school grade level needed for text comprehension. This metric considers the average sentence length (number of words per sentence) and the average number of syllables per word. The formula is (0.39 × average sentence length) + (11.8 × average syllables per word) − 15.59. The resulting score corresponds to the US grade level education needed to understand the text. This metric is particularly beneficial for ensuring that ICFs are accessible to individuals with varying levels of education, aligning with IRB guidelines.

The Gunning Fog Index [25] assesses the complexity of a text based on two variables: the average sentence length and the proportion of complex words (defined as words with three or more syllables). Its formula, 0.4 x ((average sentence length) + (percentage of complex words)), yields a score that represents the years of formal education a reader requires to understand the text on the first reading. A lower score suggests more straightforward language, increasing the text’s accessibility. This index provides an in-depth look at text complexity, highlighting areas where simplification can make the text more accessible to a broader audience.

Additionally, our analysis incorporates the textstat library, utilizing the Dale–Chall readability formula, to determine the count of challenging words in an ICF based on a standard list of 3000 words commonly known to 4th-grade students in the USA. Words not included in this list are classified as difficult. Furthermore, the formula factors in the average length of sentences and the proportion of words with three or more syllables.

### Statistical methods

Box plots were used to describe the distribution of Flesch-Kincaid Grade Level aggregated by year. We graphically represented the total number of clinical trials, the number of clinical trials surpassing the 8th-grade Flesch-Kincaid Grade Level, and the number of clinical trials surpassing the 10th-grade Flesch-Kincaid Grade Level, aggregated by year. Specific yearly data points were omitted from the early and latter parts of the dataset due to the small number of studies in those years, which could skew the interpretation of trends.

Based on a separate graphical representation percent of clinical trials with ICFs above the 8th and 9th Flesch-Kincaid Grade Levels aggregated by year, we examined longitudinal trends with a Wilcoxon-type nonparametric linear trend test [26].

The statistical analysis and figure generation used the Stata software package (version 17) [27].

### Data availability

The dataset supporting this manuscript is available in the following public data repository: http//doi.org/10.6084/m9.figshare.25137323

## Results

Our examination of 5,239 ICFs from ClinicalTrials.gov revealed several key insights regarding their readability and complexity (Table 2):

**Table 2.** Comparative analysis of readability metrics (ClinicalTrials.gov, 2005–2024, *n* = 5239)

Metric	Score	References [11]				
Flesch Reading Ease	55.13 (SD = 8.97) (range = 98.78)	Flesch Reading Ease Score	Estimated school grade completion			Estimated percent of US adults
		90–100	4th grade			93
		80–90	5th grade			91
		70–80	6th grade			88
		60–70	7 or 8th grade			83
		50–60	Some high school			54
		30–50	High school to college			33
		0–30	College			4.5
Flesch- Kincaid Grade Level	10.99 (SD = 2.45) (range = 41.3)	Flesch-Kincaid Grade Level		Education Level Correspondence		
		4–5		Intermediate elementary school (4th-5th grade)		
		6–7		Middle school (6th–7th grade)		
		8–9		Early high school (8th–9th grade)		
		10–11		Late high school (10th–11th grade)		
		12–13		College level (12th grade–1st year college)		
		14–15		College graduate level (2nd-year college–graduate)		
		16+		Professional and advanced degrees		
		Gunning Fog Index			Reading level by grade	
Gunning Fog Index	10.91 (SD = 2.80) range = 46.96)	13			College freshman	
		12			High school senior	
		11			High school junior	
		10			High school sophomore	
		9			High school freshman	
		8			8th grade	
		7			7th grade	
		6			6th grade

**Figure 2.**
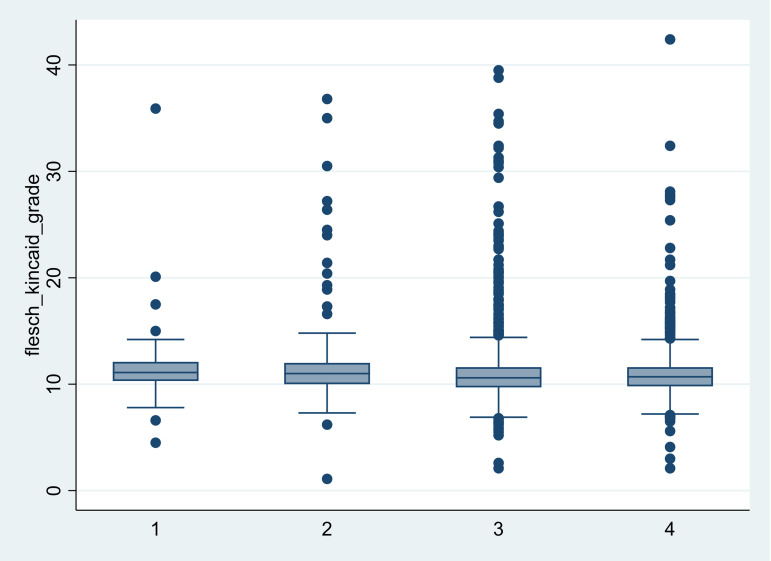
Distribution of Flesch-Kincaid Grade Level scores aggregated by year (ClinicalTrials.gov, 2005–2024, *n* = 5239).

- Flesch Reading Ease Scores: The mean score was 55.13 (SD = 8.97), indicating a moderate reading difficulty level (equivalent to a high school education level) below the commonly accepted readability range of 60–70.
- Flesch-Kincaid Grade Level: The average grade level was 10.99 (SD = 2.45) (Figure 2) exceeding the 8th-grade reading level recommended by IRB guidelines.
- Gunning Fog Index: The mean score of 10.91 (SD = 2.80) is equivalent to almost a high school junior-level grade and may be too complex for a general audience. Lower scores are preferred to enhance comprehension.
- Percentage of Difficult Words: The average proportion was 0.11 (SD = 0.05), providing further evidence of the complexity within the ICF texts.
- Document Length: The average document was 13.95 pages long (SD = 13.05) compared to the suggested maximum 10-page length.
- Readability Level Discrepancy: 91% (4,768 out of 5,239) of the ICFs were assessed to have a reading level above the 8th grade.

### Trends in readability levels of clinical studies

**Figure 3.**
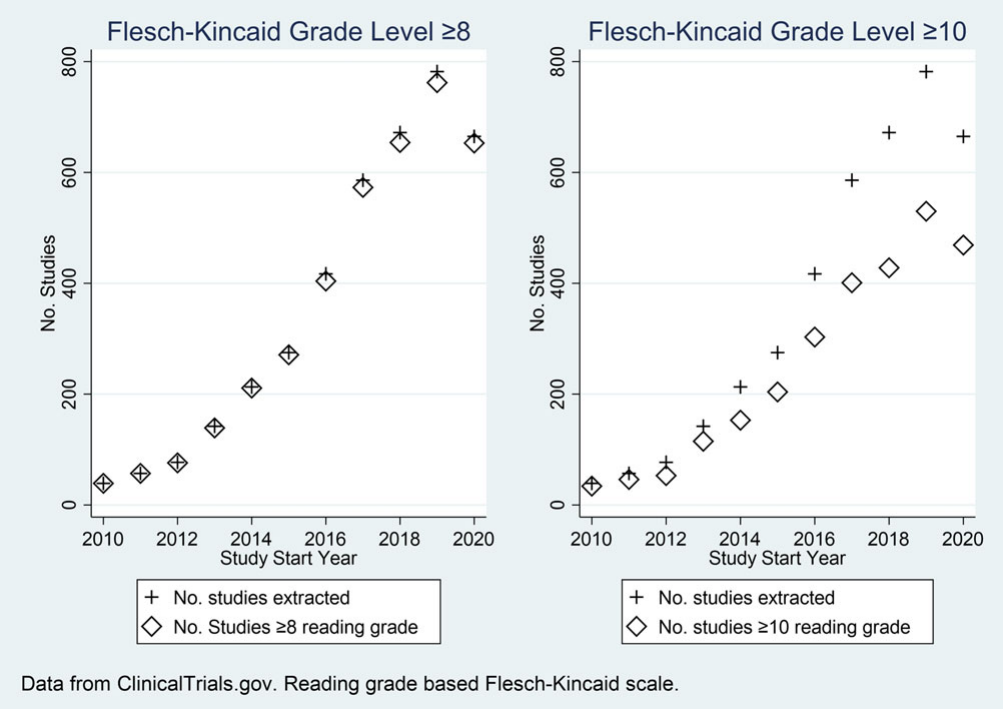
Trends in clinical study readability: comparison of studies with ≥8 vs. ≥10 Flesch-Kincaid Grade Levels (ClinicalTrials.gov, 2010–2020, *n* = 3925).

An analysis of clinical studies from ClinicalTrials.gov spanning a decade revealed changing trends in the readability levels of ICFs. Box plots revealed a stable median Flesch-Kincaid Grade Level, with an overall median (interquartile range) of 10.7 (9.98 1 11.7) and substantial outliers at the top of the scale (Figure 2). The proportion of studies with ICFs meeting a Flesch-Kincaid Grade Level of ≥ 8 remained relatively stable over time. In contrast, there was a notable downward trend in the absolute number (Figure 3) of studies with ICFs exceeding grade level of ≥ 10.

**Figure 4.**
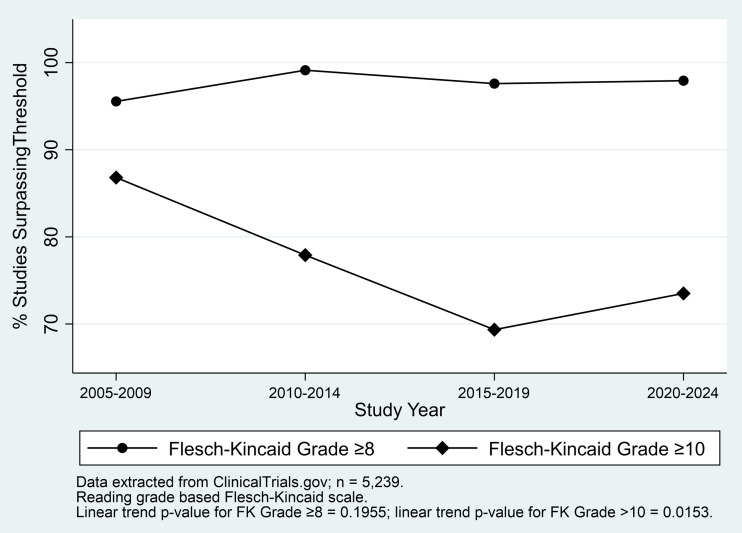
Four-year interval trends in the percent of clinical studies exceeding Flesch-Kincaid Grade Level thresholds (ClinicalTrials.gov, 2005–2024, *n* = 5,239).

Figure 4 presents a longitudinal view of the percent of studies surpassing specific Flesch-Kincaid Grade Level thresholds, grouped in 5-year intervals. There is a decrease in the percentage of studies with ICFs written at a Flesch-Kincaid Grade Level of ≥ 10 over the studied period. This downward trend, which is statistically significant with a p-value of 0.0153, indicates a positive shift toward creating more accessible ICFs over the past two decades. However, the percentage of studies with ICFs exceeding a grade level of ≥8 shows a slight fluctuation without a significant linear trend (*p* = 0.1955). This suggests that while there is an overall improvement in reducing the highest levels of readability complexity (i.e., fewer ICFs at or above the 10th-grade level), the progress in achieving readability at the recommended 8th-grade level is less consistent and still requires attention to meet standard readability guidelines.

## Discussion

Leading academic institutions, such as Johns Hopkins Medicine and Yale, emphasize the importance of maintaining the reading level of ICFs at or below the 8th grade, with some populations requiring material at the 5th- or 6th-grade level. These guidelines are crucial in ensuring that consent forms are accessible to a broader demographic, especially considering the diverse educational backgrounds of potential research participants [28,29].

The examination of 5,239 CFs from ClinicalTrials.gov highlights a significant challenge in readability, emphasizing the need to improve the informed consent process within clinical research. A prominent issue is the misalignment between the ideal 8th-grade reading level recommended by IRBs and the actual, more complex levels observed in most ICFs. This discrepancy, as indicated by the average Flesch-Kincaid Grade Levels and elevated Gunning Fog Index scores, raises concerns about the efficacy of informed consent and the potential for misunderstandings among participants.

Furthermore, the extensive length of these documents, averaging around 14 pages, poses a substantial risk of overwhelming participants and impeding their understanding. Prior research has suggested an upper limit of 10 pages for an ICF, with more extended forms, such as those spanning 25 pages, potentially requiring 34–48 minutes to read, depending on the participant’s reading level [5]. This complexity challenges the ethical integrity of the consent obtained and may limit the diversity of research participants and affect the representativeness of study outcomes. Therefore, it is essential to simplify ICF content by using more straightforward language, shortening document length, and adopting more engaging formats.

However, it is essential to recognize that while these indices provide a quantitative measure of readability, they do not fully encapsulate the multifaceted nature of comprehension [30]. Comprehension is influenced by various factors including the complexity of the information, the format and structure of the text, the cultural and educational background of the reader, and also the training of the research staff delivering the ICF and the time they spend with the participant (O’ Sullivan *et al*., 2021). Therefore, while striving to meet readability standards is essential, it is equally crucial to ensure that the content of ICFs is presented in a manner that enhances actual understanding among diverse participant populations. This may involve incorporating more qualitative methods of assessing comprehension, such as participant feedback or comprehension tests, to supplement the insights gained from readability scores. In summary, the simplification of content needs to be judiciously managed to ensure that it does not detract from the thorough understanding of the material.

While there are signs of improvement in ICF readability, particularly at grade level 10 or higher, progress remains slow. This trend indicates a growing awareness within the research community about the need for more accessible ICFs. Continuous efforts are essential to ensure informed consent reflects participants’ understanding and voluntary agreement.

This study has limitations. It focuses exclusively on US-based clinical trials and only on English-language ICFs, which may not be generalizable to international contexts or other languages. The reliance on quantitative readability scores does not fully capture the nuances of comprehension [30], and factors such as layout and design, crucial in how participants engage with and understand ICFs, were not accounted for. Another limitation is the exclusion of non-textual elements, such as diagrams or interactive components, which could influence comprehension. Lastly, because IRBs all operate independently, they may each have their own ICF template and criteria that researchers are following, or there may be other factors that influence a study’s approval (i.e. COVID-19 research) with suboptimal ICFs due to the nature of a rapid approval.

Furthermore, while quantitative readability indices provide essential measures, they do not entirely encapsulate the multifaceted nature of comprehension. Various factors, such as the complexity of the information, the format and structure of the text, and the cultural and educational background of the reader, significantly influence comprehension. The content of ICFs must be presented in a manner that enhances actual understanding among diverse participant populations, equally prioritizing qualitative assessments like participant feedback or comprehension tests to meet and surpass readability standards.

In addressing the complexity of language in ICFs, it is evident that despite the efforts of clinical translational scientists, these documents often remain mired in technical jargon and complex language structures. This issue underscores the need for employing literacy checkers and advanced technologies, such as artificial intelligence (AI), to enhance the readability of ICFs [31–34]. AI-driven language simplification tools offer a promising solution by analyzing and revising text to make it more accessible to nonspecialist audiences. However, while AI has the potential to improve the clarity of ICFs significantly, it is not infallible. The nuances of language and the critical importance of accurately conveying medical and research information necessitate a human-in-the-loop approach [35–37]. This approach ensures that AI-generated revisions are reviewed and refined by human experts, maintaining the integrity and accuracy of the information.

Furthermore, the process of refining ICF readability should not be a one-time effort but a reiterative process involving continuous human interaction. Engaging a diverse community, similar to community advisory boards [38–40] in this iterative process – what we propose as the concept of Community-in-the-Loop – can significantly enhance the relevance and effectiveness of ICFs. By incorporating feedback from a broad spectrum of stakeholders, including potential trial participants, researchers can ensure that ICFs are not only readable but also culturally and contextually appropriate.

Incorporating AI-driven language simplification techniques, with the critical oversight of human experts and the active engagement of the community, presents a comprehensive strategy to improve the accessibility of ICFs. This innovative approach, blending the capabilities of AI with the invaluable insights of diverse advisory boards and the broader community, holds significant promise for making ICFs more understandable and ethically robust. We aim to continue developing and rigorously testing these strategies, aiming to produce ICFs that meet established readability standards and facilitate informed consent in clinical research. By adopting such multifaceted and inclusive strategies, we can advance the informed consent process, making it more accessible, understandable, and, ultimately, more effective in engaging participants in clinical research.

## Conclusion

Our comprehensive review of over five thousand ICFs from ClinicalTrials.gov has revealed a crucial need for improvements in their readability. The substantial disparity between the ideal readability levels set by IRBs and the actual complexity encountered in most ICFs highlights a significant issue, potentially impacting participant comprehension and the integrity of informed consent. This study underscores the importance of simplifying ICFs to make them more accessible and understandable to diverse participant populations. Although there has been a positive trend toward improved readability, particularly at higher grade levels, much work remains. Future efforts should continue to refine these documents, ensuring legal compliance and genuine resonance with research participants. This commitment to clarity and comprehension in ICFs is fundamental to maintaining the integrity and inclusiveness of clinical research, ultimately enhancing the ethical standard of the consent process.

## Supporting information

Zai et al. supplementary material Zai et al. supplementary material

## Supplementary material

The supplementary material for this article can be found at https://doi.org/10.1017/cts.2024.595.
